# Are recent health, welfare and care graduates part of a rural and remote workforce solution? Evidence from Tasmania, Australia

**DOI:** 10.1186/s12913-024-11087-9

**Published:** 2024-05-21

**Authors:** Belinda Jessup, Fiona Proudfoot, Merylin Cross, Tony Barnett

**Affiliations:** 1 Centre for Rural Health, College of Health and Medicine, University of Tasmania, Launceston, Australia; 2 Wicking Dementia Research and Education Centre, College of Health and Medicine, University of Tasmania, Hobart, Australia

**Keywords:** Health graduate, Job vacancy, Recent graduate, Recruitment, Rural health workforce, Rural workforce solutions, Welfare graduate

## Abstract

**Background:**

Strong growth in graduate supply from health, welfare and care courses across Australia may bode well for easing rural workforce shortages. However, little is known about the employment opportunities available for recent graduates in non-metropolitan areas. This study aimed to quantify and describe advertised job vacancies for health, welfare and care professions in Tasmania, a largely rural and geographically isolated island state of Australia. Further, it aimed to examine those job vacancies specifying that recent graduates were suitable to apply.

**Methods:**

Job advertisements for health, welfare and care professionals were collected weekly throughout 2018 from six online job vacancy websites. Data were extracted on 25 variables pertaining to type of profession, number of positions, location, and graduate suitability. Location of positions were recoded into a Modified Monash Model (MM) category, the Australian geographic standard used to classify rurality. Positions advertised in MM2 areas were considered regional and MM3-7 areas rural to very remote. Data were analysed using descriptive and inferential statistics.

**Results:**

Over the twelve-month period, 3967 advertisements were identified, recruiting for more than 4700 positions across 49 different health, welfare and care professions in Tasmania. Most vacancies were in the non-government sector (58.5%) and located in regional areas (71.7%) of the state. Professions most frequently advertised were registered nurse (24.4%) and welfare worker (11.4%). Eleven professions, including physiotherapist and occupational therapist, recorded a disproportionate number of advertisements relative to workforce size, suggesting discipline specific workforce shortages. Only 4.6% of collected advertisements specified that a recent graduate would be suitable to apply. Of these, most were for the non-government sector (70.1%) and located in regional areas (73.4%). The professions of physiotherapist (26.6%) and occupational therapist (11.4%) were most frequently represented in advertised graduate suitable positions.

**Conclusions:**

Despite a range of advertised employment opportunities for health, welfare and care professionals across Tasmania, few specified vacancies as suitable for recent graduates and most were located in regional areas of the state. Health, welfare and care services in non-metropolitan locations may need to develop more employment opportunities for recent graduates and explicitly advertise these to job-seeking graduates to help grow and sustain the rural and remote health workforce into the future.

**Supplementary Information:**

The online version contains supplementary material available at 10.1186/s12913-024-11087-9.

## Background

The Australian health, welfare and care workforce has expanded rapidly over the past decade, largely due to jobs growth, the prospects of long term employment stability, and the substantial increase in graduates exiting these courses [1–5]. In part, growth in graduate numbers has been driven by projections of national workforce shortages in health, welfare and care by 2025 [6]. With the high rate of attrition of more experienced professionals [7], this expanding graduate workforce was also expected to meet the service demand given the ageing population, the growing prevalence of chronic disease and the roll out of the National Disability Insurance Scheme [8]. More recently, strong graduate supply has also been identified as a critical component of surge workforce capability [9], necessary given the unprecedented impact of the COVID-19 pandemic [10], the repercussions of natural disasters on welfare and care workforce demand, as well as the workforce mobility experienced within this sector [11].

There is emerging concern that health, welfare and care services may not be structured to cope with the record supply of graduates, with some recently qualified professionals experiencing difficulties securing initial employment [2, 3, 5, 12]. In 2021, the Graduate Outcomes Survey [13], which measures short-term employment outcomes in Australia, recorded the lowest percentage of graduates employed in some capacity at four months post-graduation (84.8%), with full-time employment at 68.9%. This report indicated that 15.2% of graduates were unemployed, and a further 15.9% were underemployed at four months post-qualifying. Although the same survey repeated in 2022 recorded an increase in both overall employment and full-time employment figures to 88.3% and 78.5% respectively, full-time employment rates varied across disciplines (e.g., 72.0% for psychology, 82.6% for nursing and 96.2% for pharmacy) [13]. Overall employment figures were also higher, irrespective of discipline, indicating that many health, welfare and care graduates experienced underemployment from having to commence their careers in part-time roles [2, 5, 12, 13]. Both unemployment and underemployment among health graduates is likely influenced by the restricted number, and hence competitive nature of graduate specific positions which offer additional support and supervision in the immediate post-graduate year [2, 12]. For some health disciplines such as nursing, psychology and pharmacy with structured, and sometimes compulsory postgraduate registrar or internship requirements, competitively gaining a post-graduate position that offers the required supervision can prove time intensive [14, 15]. A study by Wong and colleagues [15] for example showed that in Australia, 42.7% of clinical psychology and clinical neuropsychology graduates took six months or longer to commence employment in a registrar position after completing their postgraduate studies.

While an increasingly competitive labour market will concern some graduates, ongoing rural workforce shortages create key opportunities to gain initial employment [16–18]. Health, welfare and care service managers often offer graduate positions to build rural workforce capacity given they typically attract a greater applicant pool and are therefore easier to fill [19, 20]. This is fortuitous as health, welfare and care graduates are becoming increasingly motivated to seek rural employment, through affirmative selection of students with a rural background [21], positive rural clinical training experiences [22], previous experience either living or studying in that location [23], or through personal motivation to practice in a rural setting [23]. While this opportunistic employment of recent graduates motivated toward a career in rural health is congruent with sustainable rural health workforce policy [24], recent studies show a degree of ‘urban drift’ amongst rural origin health graduates, suggesting a lack of suitable positions in rural areas [25, 26]. In part, this may reflect the challenges in resourcing and supporting graduate positions in rural areas [18, 27]. Recent graduates need adequate supervision, training, opportunities for career advancement, diverse work opportunities and community integration to support positive transition into the rural workforce [18, 23, 27].

Several studies have reported the number of health, welfare and care graduates practising in rural areas post-graduation [25, 26, 28], and others quantify rural job opportunities for specific professions [20, 29, 30]. However, this literature fails to reflect more broadly the discipline areas seeking to employ graduates in non-metropolitan locations, or how many positions are available. With a growing supply of graduates potentially able to ease rural workforce shortages, this study has dual aims. First, to describe employment opportunities for health, welfare and care professions in Tasmania; a largely rural island state of Australia which faces ongoing challenges building a sustainable health workforce [31]. It was hypothesised that the number of advertisements for these professions in Tasmania would be proportionate to the size of that workforce and that if the proportions were higher, this may reflect a shortage of professionals in that area. Second, this study aimed to examine advertised vacancies specifying graduate suitability, with professions explicitly seeking to recruit recent graduates anticipated to be experiencing greater flexibility in the employment of new graduates and hence their suitability to help address a workforce shortage.

As a geographically isolated island state of Australia separated from the mainland by Bass Strait, Tasmania offers a unique context to explore the issue of workforce supply and demand. It is a relatively small island of approximately 68,401km^2^, and home to 506,736 people [32]. Tasmania’s largest population centres of Hobart, the capital city, and Launceston, are both considered regional (MM2) under the Modified Monash Model for classifying rurality in Australia, and are home to around 60% of the state’s population [32]. The remainder of Tasmania’s population are dispersed across rural and remote communities (MM3-7), with the state having the largest percentage of residents living in small rural towns (MM5) (18.2%) Australia-wide [32]. In 2016, the health, welfare and care workforce in Tasmania comprised 25,413 professionals, with nursing and carers forming the largest cohorts [33]. The workforce was largely centralised in MM2 areas. Although growth has been observed in the health, welfare and care workforce in rural and remote areas (MM3-7), there continues to be a disproportionately lower per capita distribution of health professionals in these areas [33]. The ongoing challenge of resourcing Tasmania’s health, welfare and care workforce is, in part, due to the absence of local training pathways, especially for allied health professions such as physiotherapy, occupational therapy, optometry, and podiatry, and difficulties recruiting tertiary qualified health professionals from mainland Australia [31]. The findings of this study are therefore likely to prove useful in guiding policy initiatives and strategic investment to build sustainable rural and remote workforce in Tasmania, and elsewhere.

## Methods

This study employed a repeated cross-sectional longitudinal design, with weekly collection of online job vacancy advertisements for health, welfare and care professions in Tasmania over a twelve-month period. Ethics approval was granted by the Tasmanian Human Research Ethics Committee at the University of Tasmania (H0017219).

Between 1 January 2018 and 31 December 2018, online advertisements were collected by a member of the research team (FP,BJ) for health, welfare and care professions. A broad and inclusive listing of eligible professions was compiled from a range of data sources to reflect the types of workers represented in the local Tasmanian health, welfare and care workforce [33]. In situations where there was ambiguity regarding a profession advertised, consensus discussion occurred with a second member of the research team (MC or TB) regarding inclusion.

Initially, advertisements were identified by searching 30 job vacancy data sources on the same day each week using search terms developed specifically for each source. Sources included three local newspapers, 11 online job vacancy websites and 16 professional associations related to one of the health, welfare and care professions included in this study. However, after a four-week review period, no unique advertisements were identified beyond searches conducted in six online job vacancy websites (see additional file 1). Therefore, for the remaining 11 months of the study, only these six websites were used to collect advertisements.

Included advertisements were clipped using Evernote and electronically stored. Where possible, data for 25 variables were extracted for each advertisement including: date of advertisement; number of positions vacant; health profession/s; job title/s; type of employer (government, non-government); employment contract/s (temporary versus permanent); employment tenure/s (full-time, part-time, casual); level/grade of position/s; qualifications and experience required; salary; location/s of position/s; whether the position had previously been advertised; and if applications would be used to fill subsequent or similar positions. Graduate suitability was also coded, which for the purposes of this study was when the advertisement explicitly encouraged recent graduates to apply. Position location/s were subsequently recoded using the Modified Monash Model (MM). Positions in MM2 were categorised as regional, MM3-5 as rural, and MM6-7 as remote [34]. Position/s with multiple locations across regional, rural and remote areas, or that were advertised as ‘statewide’, were categorised as ‘mixed MM2-7’.

All extracted data were analysed using IBM SPSS Statistics (Version 27) (https://www.ibm.com/products/spss-statistics). Descriptive statistics were conducted to identify sample characteristics and relationships between variables were analysed using the Chi-square statistic. Z scores were calculated between population proportions for workforce size (drawing on 2016 census data provided by the Australian Bureau of Statistics (ABS)) and advertising frequency for individual professions. A *p*-value of < 0.05 was accepted as statistically significant for all calculations.

## Results

### Total advertisements

Over the study period, a total of 3967 job advertisements were collected for health, welfare and care vacancies located across Tasmania. The second half of the year (July through December, *n* = 2082, 52.5%) yielded a significantly greater number of advertisements than the first half (January through June, *n* = 1885, 47.5%) (χ^2^ = 16.6, 3, *p* < 0.001).

While most job advertisements were for a single vacancy, 734 (18.5%) were recruiting for multiple vacancies. The total number of positions advertised exceeded 4700 (Table 1). Most advertisements recruiting for multiple vacancies were seeking registered nurses (*n* = 222, 30.2%), mixed professional backgrounds (*n* = 110, 15.0%), carers and/or aides (*n* = 97, 13.2%), or welfare workers (*n* = 66, 9.0%). Advertisements for multiple vacancies represented more than 25% of total advertisements for the professions of paramedic, midwife, carer and/or aide, dental therapist, youth justice health professional, enrolled nurse and radiographer (see additional file 2).

**Table 1:** Job advertisements for health, welfare and care professions across Tasmania (*n* = 3967)

Health, Welfare or Care Profession	Advertisements for a Single Position *n* = 3233	Advertisements for Two or More Positions *n* = 734	Total Advertisements *n* = 3967
	*n* (%)	*n* (%)	*n* (%)
Registered Nurse*	732 (22.6)	222 (30.2)	954 (24.0)
Welfare Worker	384 (11.9)	66 (9.0)	450 (11.3)
Multiple Professions Specified	310 (9.6)	115 (15.7)	425 (10.7)
Health Professional Manager	301 (9.3)	21 (2.9)	322 (8.1)
Carer and/or Aide	204 (6.3)	97 (13.2)	301 (7.6)
Physiotherapist*	203 (6.3)	30 (4.1)	233 (5.9)
Occupational Therapist*	121 (3.7)	18 (2.5)	139 (3.5)
Allied Health Assistant	104 (3.2)	12 (1.6)	116 (2.9)
Pharmacist*	95 (2.9)	14 (1.9)	109 (2.7)
Enrolled Nurse*	73 (2.3)	29 (4.0)	102 (2.6)
Psychologist*	78 (2.4)	5 (0.7)	83 (2.1)
Health Profession Project Role	79 (2.4)	3 (0.4)	82 (2.1)
Case Manager	49 (1.5)	4 (0.5)	53 (1.3)
Child and Family/Youth Justice Health Professional	34 (1.1)	19 (2.6)	53 (1.3)
Nursing Support Worker	41 (1.3)	12 (1.6)	53 (1.3)
Social Worker	46 (1.4)	0 (0.0)	46 (1.2)
Sonographer/Ultrasonographer	41 (1.3)	4 (0.5)	45 (1.1)
Counsellor	39 (1.2)	5 (0.7)	44 (1.1)
Speech Pathologist	30 (0.9)	3 (0.4)	33 (0.8)
Podiatrist*	28 (0.9)	3 (0.4)	31 (0.8)
Radiographer*	22 (0.7)	8 (1.1)	30 (0.8)
Midwife*	13 (0.4)	11 (1.5)	24 (0.6)
Optometrist*	16 (0.5)	5 (0.7)	21 (0.5)
Exercise Physiologist	20 (0.6)	0 (0.0)	20 (0.5)
Dietitian	15 (0.5)	2 (0.3)	17 (0.4)
Environmental/Public Health Officer	16 (0.5)	1 (0.1)	17 (0.4)
Diversional Therapist	14 (0.4)	2 (0.3)	16 (0.4)
Alcohol and Other Drug Worker	13 (0.4)	2 (0.3)	15 (0.4)
Paramedic*	7 (0.2)	8 (1.1)	15 (0.4)
Other	11 (0.3)	4 (0.5)	15 (0.4)
Hospital/Medical Scientist	11 (0.3)	3 (0.4)	14 (0.4)
Audiologist	12 (0.4)	1 (0.1)	13 (0.3)
Nurse Practitioner	9 (0.3)	0 (0.0)	9 (0.2)
ACAT Assessor	6 (0.2)	2 (0.3)	8 (0.2)
Aboriginal Health Worker	7 (0.2)	0 (0.0)	7 (0.2)
Radiation Therapist	6 (0.2)	1 (0.1)	7 (0.2)
Mammographer	6 (0.2)	0 (0.0)	6 (0.2)
Scientific/Research Officer	6 (0.2)	0 (0.0)	6 (0.2)
Health/Medical Physicist	5 (0.2)	0 (0.0)	5 (0.1)
Cardiology Health Professional	4 (0.1)	0 (0.0)	4 (0.1)
Complementary Health Therapist	4 (0.1)	0 (0.0)	4 (0.1)
Dental Prosthetist	3 (0.1)	1 (0.1)	4 (0.1)
Orthotist/Prosthetist	4 (0.1)	0 (0.0)	4 (0.1)
Dental Therapist	2 (0.0)	1 (0.1)	3 (0.1)
Respiratory Scientist	3 (0.1)	0 (0.0)	3 (0.1)
Microbiologist	2 (0.0)	0 (0.0)	2 (0.1)
Perfusionist	2 (0.0)	0 (0.0)	2 (0.1)
Epidemiologist	1 (0.0)	0 (0.0)	1 (0.0)
Nuclear Medicine Health Professional	1 (0.0)	0 (0.0)	1 (0.0)
Total	**3233 (100.0)**	**734 (100.0)**	**3967 (100.0)**

*AHPRA regulated profession

### Professions advertised

Advertisements were collected for a total of 49 different health, welfare and care professions. The largest number of advertisements were collected for the professions of registered nurse (*n* = 954, 24.0%), followed by welfare worker (*n* = 450, 11.4%) (Table 1). Together with vacancies that were open to applicants from multiple professions (*n* = 425, 10.7%) and health professional managers (*n* = 322, 8.1%), these four groups accounted for just over half of all job advertisements. Seventeen professions recorded less than 10 advertisements across the twelve month period.

Among the 425 (10.7%) advertisements promoting vacancies as suitable for multiple professions, up to eight different professions were listed, though most specified two (*n* = 214, 51.3%) or three (*n* = 113, 27.1%) professions (Table 2). Of the advertisements specifying two different professions, most were for combinations of: registered nurse or enrolled nurse (*n* = 61, 28.5%); occupational therapist or physiotherapist (*n* = 56, 26.2%); psychologist or counsellor (*n* = 38, 17.8%); and psychologist or social worker (*n* = 32, 15.0%). Just over three quarters (*n* = 86, 76.1%) of advertisements that listed three different professions were recruiting for combinations of: occupational therapist, physiotherapist or exercise physiologist (*n* = 42, 37.2%); occupational therapist, psychologist or social worker (*n* = 25, 22.1%); and psychologist, social worker or counsellor (*n* = 19, 16.8%).

Among the 49 professions identified, only advertisements for health professional managers and case workers listed various professions as suitable for the role. For health professional managers, up to five professions were listed; however, most advertisements specified a single profession (*n* = 310, 96.3%), predominantly a registered nurse (*n* = 200, 62.1%) (Table 2). While most advertisements for case managers also advertised for a single profession (*n* = 41, 77.4%), some listed up to four professions. Over half (*n* = 30, 56.6%) of the advertisements for case workers did not specify what profession should apply, with the remaining 23 advertisements seeking to recruit mostly social workers (*n* = 21) and psychologists (*n* = 11).

**Table 2:** Advertisements specifying suitability for different professional backgrounds

	Number of Advertisements	Maximum Number of Professions Advertised as Suitable	Number (*n*) and type of professions advertised (listed in descending order of frequency)
**All Advertisements**			
*Multiple Professions Specified*	425	8	(22) psychologist, occupational therapist, social worker, physiotherapist, registered nurse, counsellor, exercise physiologist, enrolled nurse, speech pathologist, welfare worker, dietitian, chiropractor, podiatrist, orthoptist, osteopath, radiographer, paramedic, midwife, Aboriginal health worker, mammographer, nutritionist, sonographer
*Health Professional Manager*	322	5	(13) registered nurse, pharmacists, psychologist, social worker, physiotherapist, counsellor, enrolled nurse, occupational therapist, midwife, welfare worker, speech pathologist, children and family/youth justice health worker, exercise physiologist
*Case Manager*	53	4	(5) social worker, psychologist, occupational therapist, registered nurse, welfare worker
**Graduate Suitable Advertisements**			
*Multiple Professions Specified*	33	7	(10) occupational therapist, physiotherapist, exercise physiologist, psychologist, counsellor, social worker, registered nurse, midwife, osteopath, dietitian

Based on ABS workforce data, eleven professions recorded significantly more advertisements than expected (welfare worker, health professional manager, physiotherapist, occupational therapist, nursing support worker, allied health assistant, podiatrist, sonographer, exercise physiologist, alcohol and other drug worker, cardiology health professional) (Table 3). Conversely, eight professions (carer and/or aide, enrolled nurse, social worker, midwife, counsellor, paramedic, hospital/medical scientist, complementary health therapist) showed proportionately fewer advertisements than expected.

**Table 3:** Demand for health, welfare and care professions based on significant differences between proportion of workforce and number of advertisements

Profession	ABS Code	2016 Census Headcount	% of Total Workforce	No. of Advertisements	% of Total Advertisements	z Score
**In High Demand**						
*Welfare Worker*	*272,613 Welfare Worker; 4117 Welfare Support Worker*	1867	9.8	450	13.6	-6.5047*
*Health Professional Manager*	*1342 Health and Welfare Services Managers*	553	2.9	322	9.7	-18.6006*
*Physiotherapist*	*2525 Physiotherapists*	367	1.9	233	7.0	-16.724*
*Occupational Therapist*	*2524 Occupational Therapists*	229	1.2	139	4.2	-12.451*
*Nursing Support Worker*	*423,312 Nursing Support Worker*	172	0.9	53	1.6	-3.6803*
*Allied Health Assistant*	*423,314 Therapy Aide*	137	0.7	116	3.5	-13.9289*
*Podiatrist*	*2526 Podiatrists*	90	0.5	31	0.9	-3.3325*
*Sonographer/Ultra Sonographer*	*251,214 Sonographer*	86	0.5	45	1.4	-6.2853*
*Exercise Physiologist*	*234,915 Exercise Physiologist*	49	0.3	20	0.6	-3.3006*
*Alcohol and Other Drug Worker*	*272,112 Drug and Alcohol Counsellor*	47	0.2	15	0.5	-2.0662*
*Cardiology Health Professional*	*311,212 Cardiac Technician*	3	0.0	4	0.1	-3.144*
**In Low Demand**						
*Carer and/or Aide*	*4200 carers and aides nfd; 4230 personal carers and assistants nfd; 4231 Aged and Disabled Carers*	5278	27.8	301	9.1	22.9672*
*Enrolled Nurse*	*411,411 Enrolled Nurse*	882	4.6	102	3.1	4.0617*
*Social Worker*	*2725 Social Workers*	639	3.4	46	1.4	6.0929*
*Midwife*	*2541 Midwives*	424	2.2	24	0.7	5.7169*
*Counsellor*	*2721 Counsellors*	370	1.9	44	1.3	2.4487*
*Paramedic*	*4111 Ambulance Officers and Paramedics*	322	1.7	15	0.5	5.4169*
*Hospital/Medical Scientist*	*2346 Medical Laboratory Scientist*	256	1.3	14	0.4	4.5001*
*Complementary Health Therapist*	*2522 Complementary Health Therapist*	90	0.5	4	0.1	2.8986*

*p < 0.05

### Location of job vacancies

Most advertisements (*n* = 2845, 71.7%) were for positions located in the state’s two largest population centres, Hobart and Launceston (both MM2) (Table 4). The remainder were spread across rural (MM3-5) (*n* = 693, 17.4%) and remote communities (MM6-7) (*n* = 57, 1.5%), mixed (MM2-7) locations (*n* = 51, 1.3%), or were unspecified (*n* = 321, 8.1%).

**Table 4:** Characteristics of job advertisements (*n* = 3967)

Advertisement Characteristic	Advertisements		
	Graduate Suitable *n* = 184 *n* (%)	Other *n* = 3783 *n* (%)	Total *n* = 3967 *n* (%)
**Location**			
*MM 2*	135 (73.4)	2710 (71.6)	2845 (71.7)
*MM 3–5*	22 (11.9)	671 (17.7)	693 (17.4)
*MM 6–7*	0 (0.0)	57 (1.5)	57 (1.5)
*Mixed MM 2–7*	8 (4.3)	43 (1.1)	51 (1.3)
*Not Specified*	19 (10.3)	302 (8.0)	321 (8.1)
**Employer**			
*Government*	18 (9.8)	1307 (34.5)	1325 (33.4)
*Non-Government*	129 (70.1)	2192 (57.9)	2321 (58.5)
*Not Specified*	37 (20.1)	284 (7.5)	321 (8.1)
**Tenure**			
*Permanent*	27 (14.7)	1159 (30.6)	1186 (29.9)
*Temporary/Fixed Term*	39 (21.2)	943 (24.9)	982 (24.7)
*Mixed Tenures*	3 (1.6)	129 (3.4)	132 (3.3)
*Not Specified*	115 (62.5)	1552 (41.0)	1667 (42.0)
**Hours of Employment**			
*Full-Time*	76 (41.3)	1438 (38.0)	1514 (38.2)
*Part-Time*	22 (12.0)	932 (24.6)	954 (24.1)
*Casual*	8 (4.3)	431 (11.4)	439 (11.1)
*Locum*	11 (6.0)	46 (1.2)	57 (1.4)
*Variable Hours*	33 (17.9)	457 (12.1)	490 (12.4)
*Not Specified*	34 (18.5)	479 (12.7)	513 (12.9)
**Qualification Requirements**			
*Undergraduate*	157 (85.3)	2268 (60.0)	2425 (61.1)
*Postgraduate*	1 (0.6)	62 (1.6)	63 (1.6)
*Vocational*	1 (0.6)	445 (11.8)	446 (11.3)
*Undergraduate or Vocational*	2 (1.1)	144 (3.8)	146 (3.7)
*Not Specified*	23 (12.5)	837 (22.1)	860 (21.7)
**Experience**			
*Experience Essential*	13 (7.1)	1442 (38.1)	1455 (36.7)
*Experience Desirable*	30 (16.3)	427 (11.3)	457 (11.5)
*No Experience Necessary*	0 (0.0)	5 (0.1)	5 (0.1)
*Not Specified*	141 (76.6)	1909 (50.5)	2050 0.7)

### Employment sector, tenure and hours of employment

Over half (*n* = 2321, 58.5%) of the advertisements were for positions within the non-government sector and a third (*n* = 1325, 33.4%) were government positions (Table 4). Irrespective of employer type, only 6.2% of advertisements indicated they were for a previously listed vacancy and 10.4% that applications may be used to ‘fill subsequent or similar positions’. The re-use of applicant data elicited from job advertisements for future vacancies was statistically more likely in the government than the non-government sector (χ^2^ = 64.8,2, *p* < 0.001).

Permanent positions were represented in only 1186 (29.9%) advertisements, with 982 (24.7%) temporary and the remaining 1667 (42.0%) not specified. Full-time positions were represented in 1514 (38.2%) advertisements, with 954 (24.1%) part-time and 439 (11.1%) casual. Locum positions were recorded in 57 (1.4%) advertisements and were largely recruiting physiotherapists (*n* = 23, 40.4%), occupational therapists (*n* = 10, 17.5%), or either of these two professions (*n* = 9, 15.8%). In those advertisements for full and part-time positions that specified tenure, a similar proportion indicated the vacancy was permanent (63%) versus fixed term (37%). Over 90% of all casual positions advertised were for a fixed term appointment.

### Qualifications and experience required

Almost three in four advertisements specified that a tertiary or vocational qualification was required (Table 4). Vocational qualifications were largely (n = 374, 83.9%) specified in advertisements for welfare workers, carers and/or aide, allied health assistants and enrolled nurses. Prior work experience was listed as either ‘essential’ or desirable’ in around half (*n* = 1912, 48.2%) of all advertisements. However, more than half of the advertisements for health professional manager, case manager, alcohol and other drug worker, ACAT assessor, mammographer and health/medical physicist, required previous work experience (see additional file 2). Only five advertisements, recruiting for an allied health assistant, a carer and/or aide and physiotherapist, indicated prior experience was not necessary for the role.

### Graduate suitable advertisements

Of the 3967 advertisements collected, only 184 (4.6%) indicated the vacancy was suitable for a recent graduate. A similar number of advertisements for graduate suitable positions were recorded in the first (*n* = 89, 48.4%) and second (*n* = 95, 51.6%) halves of the year.

While most advertisements for graduate suitable positions related to a single vacancy (*n* = 138), 46 (25.0%) were recruiting for multiple positions (Table 5). Advertisements seeking to fill multiple vacancies were mostly recruiting professionals of mixed disciplinary backgrounds (*n* = 17, 37.0%), physiotherapists (*n* = 7, 15.2%), registered nurses (*n* = 5, 10.9%), and midwives (*n* = 4, 8.7%). Advertisements for the professions of paramedic (100%) and registered nurse (80%) sought predominantly to fill multiple graduate suitable vacancies, while professions such as exercise physiologist, speech pathologist and radiographer only sought to fill single positions (see additional file 3).

**Table 5:** Job advertisements specifying graduate suitability (*n* = 184)

Health, Welfare or Care Profession	Advertisements for a Single Position *n* = 138	Advertisements for Multiple Positions *n* = 46	Total Graduate Suitable Advertisements *n* = 184	Proportion of Total Advertisements *n* = 3967
	*n* (%)	*n* (%)	*n* (%)	%
Physiotherapist*	42 (30.4)	7 (15.2)	49 (26.6)	21.0
Multiple Professions Specified	16 (11.6)	17 (37.0)	33 (17.9)	7.8
Occupational Therapist*	20 (14.5)	1 (2.2)	21 (11.4)	15.1
Registered Nurse*	10 (7.2)	5 (10.9)	15 (8.2)	1.6
Podiatrist*	10 (7.2)	2 (4.3)	12 (6.5)	38.7
Exercise Physiologist	7 (5.1)	0 (0.0)	7 (3.8)	35.0
Sonographer/Ultrasonographer	5 (3.6)	1 (2.2)	6 (3.3)	13.3
Pharmacist*	4 (2.9)	1 (2.2)	5 (2.7)	4.6
Midwife*	1 (0.7)	4 (8.7)	5 (2.7)	20.8
Welfare Worker	3 (2.2)	1 (2.2)	4 (2.2)	0.9
Enrolled Nurse*	2 (1.4)	2 (4.3)	4 (2.2)	3.9
Optometrist*	3 (2.2)	1 (2.2)	4 (2.2)	19.0
Speech Pathologist	3 (2.2)	0 (0.0)	3 (1.6)	9.1
Radiographer*	3 (2.2)	0 (0.0)	3 (1.6)	10.0
Paramedic*	0 (0.0)	3 (6.5)	3 (1.6)	20.0
Psychologist*	1 (0.7)	1 (2.2)	2 (1.1)	2.4
Nursing Support Worker	2 (1.4)	0 (0.0)	2 (1.1)	3.8
Child and Family/Youth Justice Health Professional	1 (0.7)	0 (0.0)	1 (0.6)	1.9
Dietitian	1 (0.7)	0 (0.0)	1 (0.6)	5.9
Environmental/Public Health Officer	1 (0.7)	0 (0.0)	1 (0.6)	5.9
Hospital/Medical Scientist	1 (0.7)	0 (0.0)	1 (0.6)	7.1
Health/Medical Physicist	1 (0.7)	0 (0.0)	1 (0.6)	20.0
Dental Therapist	1 (0.7)	0 (0.0)	1 (0.6)	33.3
Total (*n* = 184)	**138 (100.0)**	**46 (100.0)**	**184 (100.0)**	**4.6**

*AHPRA regulated profession

### Professions advertising graduate suitability

Only 23 of the 49 identified professions specified graduate suitable positions in one or more advertisements over the study period (Table 5). Over half of these advertisements were recruiting for physiotherapists (*n* = 49, 26.6%), professionals of mixed disciplinary backgrounds (*n* = 33, 17.9%), and occupational therapists (*n* = 21, 11.4%). Proportionately, podiatry (38.7%) and exercise physiology (35.0%) advertised graduate suitable positions the most. Another five professions (physiotherapist, midwife, paramedic, health/medical physicist, dental therapist) specified graduate suitability in more than 20% of advertisements.

Up to seven professions were listed as eligible to apply for graduate suitable vacancies calling for applicants of mixed disciplinary backgrounds. However, most advertisements specified two (*n* = 17, 51.5%), or three (*n* = 11, 33.3%) different eligible professions (Table 2). Of the advertisements specifying two different professions, most were for combinations of: physiotherapist and occupational therapist (*n* = 12, 70.6%), or psychologist and counsellor (*n* = 4, 23.5%). Of the advertisements specifying three different professions, most (*n* = 8, 72.7%) were recruiting for a combination of occupational therapist, physiotherapist or exercise physiologist.

### Location of graduate suitable positions

Almost three quarters (*n* = 135, 73.4%) of the graduate suitable positions advertised were in the regional centres of Hobart and Launceston (MM2) (Table 4). Only 22 (11.9%) were for positions in rural areas (MM3-5), and no graduate suitable vacancies were advertised exclusively in remote areas (MM6-7). There were, however, 8 (4.3%) positions advertised in mixed regional/rural/remote areas (MM2-7). Only 12 of the 23 professions that advertised graduate suitable positions were recruiting for vacancies in rural areas (MM3-5) (see additional file 3).

### Employment sector, tenure and hours of employment of graduate suitable positions

Most advertisements specifying graduate suitability were for the non-government sector (*n* = 129, 70.1%), with only 18 (9.8%) for Tasmanian government organisations (Table 4). Despite this low figure, over half of these government advertisements (*n* = 10, 55.6%) were seeking to fill multiple vacancies.

Almost two-thirds (*n* = 115, 62.5%) of advertisements for graduate suitable positions did not specify employment tenure. Only 27 (14.7%) related to a permanent role and less than half to a full-time position (*n* = 76, 41.3%). A further 39 (21.2%) offered temporary or fixed term contracts and 22 (12.0%) part-time positions. While nearly one in five (*n* = 33, 17.9%) of these advertisements offered variable hours of employment, a similar proportion did not specify the hours associated with the position (*n* = 34, 18.5%).

### Qualifications and experience required for graduate suitable positions

The majority (*n* = 157, 85.3%) of advertisements specifying graduate suitability stated tertiary qualifications were required. Experience was indicated as either ‘essential’ or ‘desirable’ for the role in just under a quarter of the advertisements (*n* = 43, 23.4%). No graduate suitable advertisements stated that ‘no experience was necessary’ (Table 4).

## Discussion

This study identified 3967 job advertisements recruiting for over 4700 vacancies across 49 different health, welfare and care professions in Tasmania over a twelve-month period. This likely represents a subset of all available employment opportunities within the state at the time of the study for several reasons. First, other recruitment methods such as word of mouth, professional networks and social media were not used to source vacancies [35]. Second, around a quarter of advertisements were recruiting for multiple vacancies, with those for graduate transition programs likely seeking to fill large numbers of positions [36]. Third, around 10% of advertisements, predominantly for vacancies in the government sector, specified that applications would be used to fill similar roles in the future. This suggests prospective employers were taking efforts to reduce advertising for individual vacancies, possibly reflecting the burdensome human resource delays associated with the recruitment of health, welfare and care professionals [18, 19, 27].

Despite these caveats, the present findings provide evidence of health recruitment activity, with many of the 49 professions identified in this study recording advertising volume comparative to workforce size. However, eleven professions, including several allied health disciplines such as physiotherapist, occupational therapist, podiatrist, sonographer, exercise physiologist, and allied health assistant appeared in demand, with advertising activity proportionately greater than expected. Shortages of allied health professionals, especially occupational therapists and physiotherapists, have been long-standing in Tasmania [37–39], and exacerbated by the introduction of the National Disability Insurance Scheme [40], which likely explains some of these findings. To help compensate for allied health professional shortages, there has been a targeted effort to grow the allied health assistant workforce in the state [40], which may also explain the high number of advertisements recorded for this profession in this study. In its strategic planning for future health workforce growth and sustainability, the Tasmanian Government has recognised the absence of local training pathways as a specific barrier to building the allied health workforce [31]. Therefore, the University of Tasmania, in concert with the Tasmanian Government, has developed a suite of new allied health degrees in physiotherapy, speech pathology and occupational therapy, to offer a sustainable home-grown solution to building workforce capacity in health professions experiencing persistent shortages [41]. While this approach aligns with recommendations within the literature [24], future research will need to explore the success of this home-grown solution to allied health workforce growth by determining the number of graduates produced, their mobility and participation in the state’s health workforce over time.

While it was possible to directly compare expected and actual advertising activity for specific disciplines, this study observed a notable 10% of vacancies were open to applicants from different professional backgrounds. Common recruitment combinations included: ‘registered OR enrolled nurse’; ‘occupational therapist OR physiotherapist OR exercise physiologist’; and ‘psychologist OR social worker OR counsellor’. This broader approach to advertising has been observed in other studies of online job advertisements specifically for occupational therapists [42] and counsellors [43], and reflects not only the blurring of professional roles and responsibilities among certain health professions [44], but the broadening scope of non-discipline specific roles that health graduates can fulfil [42]. What is not yet clear in the literature is how attractive these broader advertisements are to potential applicants compared to discipline specific roles. Neither is it clear the extent to which new graduates require a period of immersion in their profession in order to develop a professional identity [45], and hence may prefer positions in which their discipline is specified. Important areas for future research.

In a recent study by Beel [43], analysis of online counselling advertisements observed a pattern of vacancies seeking either ‘a psychologist OR social worker OR counsellor’, the same as observed in this study, and suggested that this approach enabled a broader pool of applicants for positions. Others have described this advertising behaviour as ‘workforce substitution’, and is considered an effective way of filling workforce shortages when there is a relative oversupply of one health profession compared with another [46]. In the context of this study, most professionals sought in advertisements open to different disciplines were able to be trained within the state, except for occupational therapists and physiotherapists, suggesting that an issue may be that of undersupply at a local level. For example, Tasmania has a lower number of psychologists per capita compared to other mainland states and territories, despite training pathways for this discipline being available at the University of Tasmania [31]. Positively, the University of Tasmania has recently expanded its course offerings for psychology training to the north of the state, which may help build workforce capacity in this specific discipline [47]. It will be important for future research to explore the number of graduates trained in the state and their career pathways, including post-graduate work locations over time, to understand how Tasmania can better achieve a balance between local health graduate supply and workforce demand.

Less than 5% of job advertisements specified graduate suitability, with these vacancies being largely in the non-government sector. Further, only 23 of the 49 identified health, welfare and care professions advertised graduate suitable positions. Although this overall low figure and spread across professions provides evidence of limited online recruitment activity specifically for graduates, it is important to consider not all graduate suitable positions may have been captured in this study. Tasmanian employers were potentially using other advertising methods, including word of mouth, social media and clinical placements, to recruit recent graduates directly [17, 35], especially for those professions trained within the state. Further, it is possible that online advertising activity underestimates graduate demand for some professions, with up to a quarter of graduate suitable advertisements recruiting for multiple vacancies. For professions such as midwifery, nursing and paramedicine who were found to advertise infrequently, but for many vacancies, this likely reflects graduate transition programs. For registered and enrolled nurses, such programs can offer up to 240 transition to practice positions across the state [36]. This infrequent advertising of large-scale graduate recruitment highlights a potential barrier to employment in these professions if graduates miss seeing the online advertisement, more so for interstate graduates who may not be made aware of positions by their host training university. Students in their final years and soon to graduate should obtain graduate recruitment process timelines across different jurisdictions to ensure that they do not miss out on available employment opportunities, especially for health professions with an oversupply of graduates that may require a move interstate to secure initial work [2, 12, 17].

Online advertising of graduate suitable vacancies was most common for physiotherapists, occupational therapists, or combinations thereof. Indeed, one in five of all advertised positions for physiotherapists, and over a third for podiatrists and exercise physiologists specifically sought to recruit recent graduates. This highlights the opportunities available to graduates from some allied health professions in the Tasmanian labour market and the need to reorient local employers toward how best to secure applicants for available vacancies [48]. In 2023, the Tasmanian Government began offering a scholarship program specifically targeting allied health disciplines to both attract and retain graduates to work within the state [49]. This scholarship offers students in their final year of study of select allied health disciplines a cash payment, with a second payment provided following three years service in a graduate suitable position within the state’s health workforce [49]. However, the effectiveness of this approach may be limited, with this study illustrating few graduate suitable positions to apply for. It may be a better approach to develop graduate transition programs for health, welfare and care professions in high demand such as physiotherapy and occupational therapy, and in collaboration with the non-government sector, to streamline recruitment efforts. Utilising the resources of rural workforce agencies available in each state and who are designated to support the recruitment of health, welfare and care professionals to rural areas to advertise such programs, may be an effective way to market opportunities to a broader local and interstate audience. Given the often broader nature of job advertisements noted in this study, this approach would limit wasted efforts by recent graduates who apply for positions never designed to be filled by less experienced health professionals [16, 50]. Further, it would minimise administrative burden involved in recruitment and the delays which can impact on filling roles, especially in rural areas [19, 27].

Of the graduate suitable positions identified, most were centralised in regional areas (MM2), with just over 10% located in rural towns (MM3-5), and none in remote areas (MM6-7) of the state. This finding provides some explanation for the ‘urban drift’ of rural origin health graduates toward metropolitan areas [25, 26], and suggests that rural areas may lack sufficient opportunities to cater for the number of graduates who may be motivated to seek rural employment [22]. In the Tasmanian context, rural employment opportunities may be constrained by the state’s hub and spoke approach to healthcare delivery, with more complex health services centralised in the larger regional population centres to ensure safety and quality [51]. While some services are decentralised in rural communities when safe and sustainable, this often requires a health workforce that is generalist and experienced [51]. This may challenge sustainable long term rural workforce development which emphasises the importance of recruiting graduates directly into rural positions [24]. It is acknowledged that graduate employment is resource intensive and challenging within rural contexts where funding or population size often limit the only sustainable option to sole-practice positions. This can prohibit the provision of important transitional supports including clear orientation processes, regular career check-in and development sessions, collegial practice, senior staff supervision, and social connectedness [24, 27, 52, 53]. The autonomy required for practice in more remote locations can also be challenging for recent graduates [27, 53], and may explain why around a quarter of advertisements indicating graduate suitability specified experience as necessary or desirable in this study.

With growth in the number of health graduates motivated to seek rural employment [22], the lack of rural employment opportunities suitable for graduates is a missed opportunity for sustainable rural health workforce growth in Tasmania [24]. Greater attention and collaboration are therefore needed between government and non-government sectors, and regional and rural areas of the state to develop more employment opportunities suitable for recent health, welfare and care graduates that are structured to support transition to the workforce and promote longer-term retention. These opportunities should aim to be located outside of the larger regional centres and could include cross sector roles which combine work in hospital, primary care, aged care, disability and education settings to support sustainability; positions likely to appeal to graduates given their motivation toward the acquisition of a broad range of skills and experience [18, 27, 52, 53]. However, graduates are vulnerable in their initial post-graduate years, and any positions created must seek to provide adequate supervision and training, regular access to more experienced professionals for orientation, guidance, ongoing support and mentorship, and opportunities to access quality professional development [27, 52–54]. This may require enhanced mentor recruitment, recognition and reward strategies to ensure supervisory capacity within existing rural health workforce can be achieved. Ensuring supportive management and workplace culture, the development of professional and interprofessional relationships, especially for sole positions, and financial security through full-time, permanent employment, will also be important for fostering retention [27, 28, 52, 53]. However, there is a need to ensure career pathways are available in rural settings that would allow graduates to remain in place after their completion of graduate positions [24, 53, 54].

The Tasmanian Government has already implemented strategic measures to address congoing health workforce shortages by investing in local training opportunities where sustainable to do so [31, 41], and introducing scholarship schemes to attract candidates from interstate courses [49]. While these strategies align with evidence-based approaches to rural workforce growth and sustainability [23, 54, 55], other measures are recommended including: affirmative selection of Tasmanian rural origin students into health courses given these students are more likely to remain in the state and work in rural areas post-qualifying [17, 21, 23, 54, 55]; and building high-quality placement capacity across the state, especially for allied health professions and those without local training options, to build a pipeline of interested graduates [22, 23, 54] and promote job satisfaction among the existing workforce [56]. Improving rural curricula across health courses and building generalist capabilities will also support health graduates to transition successfully into rural positions when available [27, 53, 55].

Finally, despite the ongoing nature of rural health workforce shortages and the opportunities graduate recruitment can provide to fill gaps, there is a current lack of centralised, comprehensive data to enable collective evaluation and planning of solutions [46]. It may be that a comprehensive, integrated national tracking system is needed to determine the number of new health graduates ready to enter the workforce, their pathways into employment, and where they practice over time. Although some of this information is gathered through the Graduate Outcomes Survey [13], and the Australian Health Practitioner Regulation Agency (AHPRA), both have limitations in the scope of participation of health graduates [13, 31]. The Nursing and Allied Health National Graduate Outcomes Tracking (NAHGOT) study is a coordinated effort to integrate these existing datasets to develop a more comprehensive graduate tracking system for the purposes of evaluating rural workforce participation [57]. Increased participation in NAHGOT by all University Departments of Rural Health would be a step toward improving the national picture of health graduate completions, recruitment and retention, especially in rural and remote settings. However, beyond this, continued efforts towards building a centralised, comprehensive dataset is needed to better understand the extent of health workforce shortages nationally.

There are inherent limitations with the use of online advertisements to explore health employment opportunities [58]. In addition to the limitations already described, this study may have inadvertently counted job advertisements more than once where they were advertised in different formats across the six websites. Although efforts were undertaken to match advertisement information across the different web sources from week to week to count job advertisements only once during their advertised period, this possibility cannot be discounted. The total advertisements figure may have also included readvertised job vacancies that were not filled through initial advertising efforts. Although this may have inflated the actual number of positions available over the study period, repeat advertisements profiled those professions more likely experiencing a greater workforce shortage and an inability to fill positions. Finally, advertisements for multiple vacancies were interpreted as offering two or more positions. However, as illustrated with the advertisements for graduate transition programs in some professions, including nursing [36], this may have substantially underreported the number of positions available.

## Conclusions

More than 4,700 advertised job vacancies for 49 different health, welfare and care professions were identified in Tasmania across a twelve-month period. While most disciplines recorded levels of online advertising activity consistent with workforce size, eleven professions, including physiotherapist, occupational therapist, podiatrist, sonographer, exercise physiologist and allied health assistant, appeared in demand. Most of these eleven professions were allied health disciplines and were unable to be trained within the state at the time of this study, highlighting the importance of local training pathways to support health workforce growth and sustainability in rural settings such as Tasmania.

Fewer than 5% of job advertisements specified graduates were suitable to apply, with most seeking to recruit physiotherapists, occupational therapists, or a combination thereof. Most vacancies were in the two large regional centres of the state, with no employers in remote areas, and few in rural settings, advertised employment opportunities that were suitable for recent graduates to apply. This highlights the missed opportunities to foster long term rural workforce growth by offering more positions in rural settings for rurally motivated recent health graduates.

Collaborative efforts are needed between both government and non-government health sectors, and between regional, rural and remote settings of the state to harness graduates as a rural and remote workforce solution by creating more graduate positions within healthcare organisations or networks, and introducing local graduate transition support programs through enhanced mentor recruitment, recognition and reward. The development of career pathways in rural and remote areas are also needed to complement graduate recruitment, thus allowing opportunities for those who have completed their initial transition to practice program to be retained, and hence contribute both to the local health service and community longer-term. Finally, consideration is needed for a comprehensive, integrated national tracking system to monitor health graduate numbers, their pathways into employment, and where they practice over time to help support health workforce planning at both the state and national level.

### Electronic supplementary material

Below is the link to the electronic supplementary material.

Supplementary Material 1. Additional File 1. Word document. Data sources used to identify job advertisements. Table showing search strategy for job advertisements

Supplementary Material 2. Additional File 2. Word document. Proportion of job advertisements for health, welfare and care professions across Tasmania by advertisement characteristic (*n* = 3967). Detailed analysis of job advertisements by characteristics.

Supplementary Material 3. Additional File 3. Word document. Proportion of graduate suitable job advertisements by advertisement characteristic (*n* = 184). Detailed analysis of graduate suitable job advertisements by characteristics.

## Data Availability

The datasets used and/or analysed during the current study are available from the corresponding author on reasonable request.
