# Supplementary material for: Are recent health, welfare and care graduates part of a rural and remote workforce solution? Evidence from Tasmania, Australia
Source: BMC Health Serv Res. 2024 May 21;24:652. doi: 10.1186/s12913-024-11087-9 (PMC11110370; doi:10.1186/s12913-024-11087-9)
Supplement: Supplementary file 1 — Supplementary Material 1. Additional File 1. Word document. Data sources used to identify job advertisements. Table showing search strategy for job advertisements [file 12913_2024_11087_MOESM1_ESM.docx]

Additional File 1. Data sources used to identify job advertisements

| Data Source | Name | Link Address | Search Terms |
| --- | --- | --- | --- |
| Newspaper | The Advocate | <http://www.theadvocate.com.au/classifieds/notices/> | All positions vacant |
| Newspaper | The Examiner | <http://www.examiner.com.au/classifieds/notices/> | All positions vacant |
| Newspaper | The Mercury | - | All positions vacant |
| Website | Adzuna* | [https://www.adzuna.com.au](https://www.adzuna.com.au/) | what – healthcare; where - Tasmania |
| Website | Australian JobSearch | <http://www.jobsearch.gov.au> | what – health & medical; where - Tasmania |
| Website | Careerone | [https://www.careerone.com.au](https://www.careerone.com.au/) | what – health, medical & pharmaceutical; what – voluntary, charity & social work; where - Tasmania |
| Website | Careerspot | [http://www.careerspot.com.au](http://www.careerspot.com.au/) | category – healthcare & medical; location - Tasmania |
| Website | Indeed* | <https://au.indeed.com/jobs> | what - healthcare; where - Tasmania |
| Website | Jobtome | [https://au.jobtome.com](https://au.jobtome.com/) | category – healthcare/nursing, pharmaceutical/science, social care/childcare; location – Tasmania |
| Website | Jora* | https://au.jora.com | what – healthcare; where - Tasmania |
| Website | Seek* | [www.seek.com.au](http://www.seek.com.au) | what - healthcare & medical; where - Tasmania |
| Website | Tasmanian State Government Job Vacancy Website* | [www.jobs.tas.gov.au](http://www.jobs.tas.gov.au) | job region – statewide; category – allied health; ambulance/health transport; dental; health/hospital; nursing |
| Website | Gumtree* | [www.gumtree.com.au](http://www.gumtree.com.au) | Healthcare and nursing positions vacant |
| Website | HR+ | https://www.hrplustas.com.au | Nursing and allied health positions vacant |
| Professional Association | Australian and New Zealand Society of Respiratory Science Ltd | https:// [www.anzsrs.org.au](http://www.anzsrs.org.au) | Positions vacant |
| Professional Association | Australian Association of Social Workers | <https://aasw.asn.au> | Positions vacant |
| Professional Association | Australian Community Workers Association | https://www.acwa.org.au | Positions vacant |
| Professional Association | Australian Dental Prosthetists Association | [www.adpa.com.au](http://www.adpa.com.au) | Positions vacant |
| Professional Association | Australasian Epidemiological Association | <https://aea.asn.au> | Positions vacant |
| Professional Association | Australian Institute of Medical Scientists | <https://www.aims.org.au> | Positions vacant |
| Professional Association | Australian Music Therapy Association | https://www.austmta.org.au | Positions vacant |
| Professional Association | Australian Orthotic Prosthetic Association | <https://www.aopa.org.au> | Positions vacant |
| Professional Association | Australian Physiotherapy Association | <https://www.physiotherapy.asn.au> | Positions vacant |
| Professional Association | Australian Podiatry Association | https://www.apodc.com.au | Positions vacant |
| Professional Association | Australian Psychological Society | [www.psychology.org.au](http://www.psychology.org.au) | Positions vacant |
| Professional Association | Australian Society of Medical Imaging and Radiation Therapy | [www.asmirt.org](http://www.asmirt.org) | Positions vacant |
| Professional Association | Environmental Health Professions Australia | <https://ehpa.org.au> | Positions vacant |
| Professional Association | Occupational Therapy Australia | https://www.otaus.com.au | Positions vacant |
| Professional Association | Pharmaceutical Society of Australia | <https://www.psa.org.au> | Positions vacant |
| Professional Association | Speech Pathology Australia | <https://www.speechpathologyaustralia.org.au> | Positions vacant |

*data sources used for full study period
