# Supplementary material for: Are recent health, welfare and care graduates part of a rural and remote workforce solution? Evidence from Tasmania, Australia
Source: BMC Health Serv Res. 2024 May 21;24:652. doi: 10.1186/s12913-024-11087-9 (PMC11110370; doi:10.1186/s12913-024-11087-9)
Supplement: Supplementary file 2 — Supplementary Material 2. Additional File 2. Word document. Proportion of job advertisements for health, welfare and care professions across Tasmania by advertisement characteristic (n = 3967). Detailed analysis of job advertisements by characteristics. [file 12913_2024_11087_MOESM2_ESM.docx]

Additional File 2. Proportion of job advertisements for health, welfare and care professions across Tasmania by advertisement characteristic (n=3967)

| Profession | Adverts | Advertisement Characteristic | | | | | | | | | | | | | | | | | | |
| --- | --- | --- | --- | --- | --- | --- | --- | --- | --- | --- | --- | --- | --- | --- | --- | --- | --- | --- | --- | --- |
|  |  | **Positions** | | **Employer** | | | **Tenure** | | | | **Hours** | | | | **Experience** | | | **Location** | | |
|  |  | Single | Multiple | Govt | Non-Govt | Not Specified | | Permanent | Temporary/ Fixed Term | Mixed/  Not Specified | Full-Time | Part-Time | Casual/  Locum | Mixed/  Not Specified | Essential | Desirable | Not Specified | MM2 | MM3-7 | Mixed/  Not Specified |
|  | **n** | **n (%)** | **n (%)** | **n (%)** | **n (%)** | **n (%)** | | **n (%)** | **n (%)** | **n (%)** | **n (%)** | **n (%)** | **n (%)** | **n (%)** | **n (%)** | **n (%)** | **n (%)** | **n (%)** | **n (%)** | **n (%)** |
| Registered Nurse | **954** | 732 (76.7) | 222 (23.3) | 392 (41.1) | 496 (52.0) | 66 (6.9) | | 405 (42.5) | 187 (19.6) | 362 (37.9) | 252 (26.4) | 316 (33.1) | 104 (10.9) | 282 (29.6) | 395 (41.4) | 122 (12.8) | 437 (45.8) | 678 (71.1) | 219 (23.0) | 57 (6.0) |
| Welfare Worker | **450** | 384 (85.3) | 66 (14.7) | 108 (24.0) | 320 (71.1) | 22 (4.9) | | 84 (18.7) | 162 (36.0) | 204 (45.3) | 228 (50.7) | 123 (27.3) | 42 (9.3) | 57 (12.7) | 203 (45.1) | 35 (7.8) | 212 (47.1) | 344 (76.4) | 70 (15.6) | 36 (8.0) |
| Multiple Professions Specified | **425** | 310 (72.9) | 115 (27.1) | 45 (10.6) | 323 (76.0) | 57 (13.4) | | 61 (14.4) | 93 (21.9) | 271 (63.8) | 175 (41.2) | 62 (14.6) | 50 (11.8) | 138 (32.5) | 193 (45.4) | 72 (16.9) | 160 (37.6) | 320 (75.3) | 60 (14.1) | 45 (10.6) |
| Health Professional Manager | **322** | 301 (93.5) | 21 (6.5) | 148 (46.0) | 146 (45.3) | 28 (8.7) | | 134 (41.6) | 30 (9.3) | 158 (49.1) | 205 (63.7) | 37 (11.5) | 6 (1.9) | 74 (23.0) | 168 (52.2) | 17 (5.3) | 137 (42.5) | 218 (67.7) | 72 (22.4) | 32 (9.9) |
| Carer and/or Aide | **301** | 204 (67.8) | 97 (32.2) | 27 (9.0) | 251 (83.4) | 23 (7.6) | | 34 (11.3) | 97 (32.2) | 170 (56.5) | 8 (2.7) | 72 (23.9) | 135 (44.9) | 86 (28.6) | 75 (24.9) | 46 (15.3) | 178 (59.1) | 180 (59.8) | 88 (29.2) | 33 (11.0) |
| Physiotherapist | **233** | 203 (87.1) | 30 (12.9) | 68 (29.2) | 127 (54.5) | 38 (16.3) | | 69 (29.6) | 53 (22.7) | 111 (47.6) | 100 (42.9) | 30 (12.9) | 27 (11.6) | 76 (32.6) | 38 (16.3) | 20 (8.6) | 174 (74.7) | 178 (76.4) | 31 (13.3) | 24 (10.3) |
| Occupational Therapist | **139** | 121 (87.1) | 18 (12.9) | 34 (24.5) | 88 (63.3) | 17 (12.2) | | 31 (22.3) | 29 (20.9) | 79 (56.8) | 65 (46.8) | 21 (15.1) | 14 (10.0) | 39 (28.1) | 41 (29.5) | 15 (10.8) | 83 (59.7) | 111 (79.9) | 20 (14.4) | 8 (5.8) |
| Allied Health Assistant | **116** | 104 (89.7) | 12 (10.3) | 29 (25.0) | 79 (68.1) | 8 (6.9) | | 29 (25.0) | 25 (21.6) | 62 (53.4) | 55 (47.4) | 22 (19.0) | 18 (15.5) | 21 (18.1) | 50 (43.1) | 17 (14.7) | 47 (40.5) | 93 (80.2) | 16 (13.8) | 7 (6.0) |
| Pharmacist | **109** | 95 (87.2) | 14 (12.8) | 39 (35.8) | 66 (60.6) | 4 (3.7) | | 41 (37.6) | 29 (26.6) | 39 (35.8) | 44 (40.4) | 28 (25.7) | 11 (10.1) | 26 (23.9) | 31 (28.4) | 11 (10.1) | 67 (61.5) | 77 (70.6) | 22 (20.2) | 10 (9.2) |
| Enrolled Nurse | **102** | 73 (71.6) | 29 (28.4) | 46 (45.1) | 51 (50.0) | 5 (4.9) | | 39 (38.2) | 21 (20.6) | 42 (39.6) | 19 (18.6) | 39 (38.2) | 21 (20.6) | 23 (22.5) | 23 (22.5) | 14 (13.7) | 65 (63.7) | 64 (62.7) | 36 (35.3) | 2 (2.0) |
| Psychologist | **83** | 78 (94.0) | 5 (6.0) | 45 (54.2) | 30 (36.1) | 8 (9.6) | | 33 (39.8) | 23 (27.7) | 27 (32.5) | 34 (41.0) | 25 (30.1) | 3 (3.6) | 21 (25.3) | 32 (38.6) | 8 (9.6) | 43 (51.8) | 55 (66.3) | 16 (19.3) | 12 (14.5) |
| Health Profession Project Role | **82** | 79 (96.3) | 3 (3.7) | 59 (72.0) | 20 (24.4) | 3 (3.7) | | 10 (12.2) | 60 (73.2) | 12 (14.6) | 53 (64.6) | 17 (20.7) | 8 (9.8) | 4 (4.9) | 19 (23.2) | 12 (14.6) | 51 (62.2) | 67 (81.7) | 3 (3.7) | 12 (14.6) |
| Case Manager | **53** | 49 (92.5) | 4 (7.5) | 3 (5.7) | 47 (88.7) | 3 (5.7) | | 10 (18.9) | 16 (30.2) | 27 (50.9) | 29 (54.7) | 9 (17.0) | 2 (3.8) | 13 (24.5) | 32 (60.4) | 10 (18.9) | 11 (20.8) | 44 (83.0) | 6 (11.3) | 3 (5.7) |
| Children and Families/Youth Justice Health Professional | **53** | 34 (64.2) | 19 (35.8) | 40 (75.5) | 13 (24.5) | 0 (0.0) | | 22 (41.5) | 16 (30.2) | 15 (28.3) | 28 (52.8) | 11 (20.8) | 0 (0.0) | 14 (26.4) | 12 (22.6) | 6 (11.3) | 35 (66.0) | 25 (47.2) | 6 (11.3) | 22 (41.5) |
| Nursing Support Worker | **53** | 41 (77.4) | 12 (22.6) | 36 (67.9) | 16 (30.2) | 1 (1.9) | | 31 (58.5) | 11 (20.8) | 11 (20.8) | 9 (17.0) | 24 (45.3) | 14 (26.4) | 6 (11.3) | 6 (11.3) | 10 (18.9) | 37 (69.8) | 47 (88.7) | 6 (11.3) | 0 (0.0) |
| Social Worker | **46** | 46 (100.0) | 0 (0.0) | 31 (67.4) | 15 (32.6) | 0 (0.0) | | 21 (45.7) | 20 (43.5) | 5 (10.9) | 26 (56.5) | 16 (34.8) | 2 (4.3) | 2 (4.3) | 8 (17.4) | 3 (6.5) | 35 (76.1) | 29 (63.0) | 9 (19.6) | 8 (17.4) |
| Sonographer/  Ultrasonographer | **45** | 41 (91.1) | 4 (8.9) | 6 (13.3) | 32 (71.1) | 7 (15.6) | | 10 (22.2) | 11 (24.4) | 24 (53.3) | 11 (24.4) | 5 (11.1) | 11 (24.4) | 18 (40.0) | 13 (28.9) | 3 (6.7) | 29 (64.4) | 37 (82.2) | 2 (4.4) | 6 (13.3) |
| Counsellor | **44** | 39 (88.6) | 5 (11.4) | 13 (29.5) | 25 (56.8) | 6 (13.6) | | 14 (31.8) | 17 (38.6) | 13 (29.5) | 17 (38.6) | 14 (31.8) | 1 (2.3) | 12 (27.3) | 19 (43.2) | 4 (9.1) | 21 (47.7) | 28 (63.6) | 8 (18.2) | 8 (18.2) |
| Speech Pathologist | **33** | 30 (90.9) | 3 (9.1) | 15 (45.5) | 14 (42.4) | 4 (12.1) | | 9 (27.3) | 11 (33.3) | 13 (39.4) | 9 (27.3) | 11 (33.3) | 1 (3.0) | 12 (36.4) | 7 (21.2) | 3 (9.1) | 23 (69.7) | 17 (51.5) | 9 (27.3) | 7 (21.2) |
| Podiatrist | **31** | 28 (90.3) | 3 (9.7) | 4 (12.9) | 25 (80.6) | 2 (6.5) | | 5 (16.1) | 4 (12.9) | 22 (71.0) | 14 (45.2) | 5 (16.1) | 1 (3.2) | 11 (35.5) | 4 (12.9) | 1 (3.2) | 26 (83.9) | 19 (61.3) | 5 (16.1) | 7 (22.6) |
| Radiographer | **30** | 22 (73.3) | 8 (26.7) | 16 (53.3) | 10 (33.3) | 4 (13.3) | | 14 (46.7) | 6 (20.0) | 10 (33.3) | 17 (56.7) | 3 (10.0) | 3 (10.0) | 7 (23.3) | 11 (36.7) | 0 (0.0) | 19 (63.3) | 26 (86.7) | 2 (6.7) | 2 (6.7) |
| Midwife | **24** | 13 (54.2) | 11 (45.8) | 12 (50.0) | 10 (41.7) | 2 (8.3) | | 9 (37.5) | 7 (29.2) | 8 (33.3) | 2 (8.3) | 7 (29.2) | 1 (4.2) | 14 (58.3) | 8 (33.3) | 0 (0.0) | 16 (66.7) | 19 (79.2) | 4 (16.7) | 1 (4.2) |
| Optometrist | **21** | 16 (76.2) | 5 (23.8) | 0 (0.0) | 21 (100.0) | 0 (0.0) | | 7 (33.3) | 1 (4.8) | 13 (61.9) | 12 (57.1) | 2 (9.5) | 1 (4.8) | 6 (28.6) | 9 (42.9) | 2 (9.5) | 10 (47.6) | 11 (52.4) | 5 (23.8) | 5 (23.8) |
| Exercise Physiologist | **20** | 20 (100.0) | 0 (0.0) | 1 (5.0) | 18 (90.0) | 1 (5.0) | | 2 (10.0) | 0 (0.0) | 18 (90.0) | 6 (30.0) | 5 (25.0) | 0 (0.0) | 9 (45.0) | 4 (20.0) | 2 (10.0) | 14 (70.0) | 18 (90.0) | 2 (10.0) | 0 (0.0) |
| Dietitian | **17** | 15 (88.2) | 2 (11.8) | 9 (52.9) | 6 (35.3) | 2 (11.8) | | 4 (23.5) | 6 (35.3) | 7 (41.2) | 4 (23.5) | 10 (58.8) | 0 (0.0) | 3 (17.6) | 3 (17.6) | 3 (17.6) | 11 (64.7) | 9 (52.9) | 5 (29.4) | 3 (17.6) |
| Environmental/Public Health Officer | **17** | 16 (94.1) | 1 (5.9) | 14 (82.4) | 3 (17.6) | 0 (0.0) | | 7 (41.2) | 5 (29.4) | 5 (29.4) | 15 (88.2) | 1 (5.9) | 1 (5.9) | 0 (0.0) | 3 (17.6) | 1 (5.9) | 13 (76.5) | 12 (70.6) | 4 (23.5) | 1 (5.9) |
| Diversional Therapist | **16** | 14 (87.5) | 2 (12.5) | 4 (25.0) | 12 (75.0) | 0 (0.0) | | 1 (6.3) | 3 (18.8) | 12 (75.0) | 5 (31.3) | 7 (43.8) | 4 (25.0) | 0 (0.0) | 6 (37.5) | 2 (12.5) | 8 (50.0) | 10 (62.5) | 6 (37.5) | 0 (0.0) |
| Alcohol and Other Drug Worker | **15** | 13 (86.7) | 2 (13.3) | 2 (13.3) | 12 (80.0) | 1 (6.7) | | 1 (6.7) | 2 (13.3) | 12 (80.0) | 4 (26.7) | 3 (20.0) | 4 (26.7) | 4 (26.7) | 10 (66.7) | 4 (26.7) | 1 (6.7) | 11 (73.3) | 4 (26.7) | 0 (0.0) |
| Paramedic | **15** | 7 (46.7) | 8 (53.3) | 15 (100.0) | 0 (0.0) | 0 (0.0) | | 5 (33.3) | 4 (26.7) | 6 (40.0) | 9 (60.0) | 1 (6.7) | 0 (0.0) | 5 (33.3) | 4 (26.7) | 0 (0.0) | 11 (73.3) | 4 (26.7) | 1 (6.7) | 10 (66.7) |
| Other | **15** | 11 (73.3) | 4 (26.7) | 5 (33.3) | 7 (46.7) | 3 (20.0) | | 2 (13.3) | 6 (40.0) | 7 (46.7) | 5 (33.3) | 4 (26.7) | 3 (20.0) | 3 (20.0) | 4 (26.7) | 3 (20.0) | 8 (53.3) | 11 (73.3) | 1 (6.7) | 3 (20.0) |
| Hospital/Medical Scientist | **14** | 11 (78.6) | 3 (21.4) | 12 (85.7) | 1 (7.1) | 1 (7.1) | | 6 (42.9) | 7 (50.0) | 1 (7.1) | 5 (35.7) | 8 (57.1) | 1 (7.1) | 0 (0.0) | 0 (0.0) | 2 (14.3) | 12 (85.7) | 13 (92.9) | 0 (0.0) | 1 (7.1) |
| Audiologist | **13** | 12 (92.3) | 1 (7.7) | 2 (15.4) | 10 (76.9) | 1 (7.7) | | 4 (30.8) | 2 (15.4) | 7 (53.8) | 5 (38.5) | 3 (23.1) | 0 (0.0) | 5 (38.5) | 6 (46.2) | 0 (0.0) | 7 (53.8) | 8 (61.5) | 5 (38.5) | 0 (0.0) |
| Nurse Practitioner | **9** | 9 (100.0) | 0 (0.0) | 6 (66.7) | 2 (22.2) | 1 (11.1) | | 6 (66.7) | 2 (22.2) | 1 (11.1) | 7 (77.8) | 2 (22.2) | 0 (0.0) | 0 (0.0) | 2 (22.2) | 0 (0.0) | 7 (77.8) | 6 (66.7) | 2 (22.2) | 1 (11.1) |
| ACAT Assessor | **8** | 6 (75.0) | 2 (25.0) | 4 (50.0) | 4 (50.0) | 0 (0.0) | | 2 (25.0) | 5 (62.5) | 1 (12.5) | 0 (0.0) | 1 (12.5) | 4 (50.0) | 3 (37.5) | 4 (50.0) | 1 (12.5) | 3 (37.5) | 5 (62.5) | 2 (25.0) | 1 (12.5) |
| Aboriginal Health Worker | **7** | 7 (100.0) | 0 (0.0) | 5 (71.4) | 2 (28.6) | 0 (0.0) | | 2 (28.6) | 3 (42.9) | 2 (28.6) | 5 (71.4) | 2 (28.6) | 0 (0.0) | 0 (0.0) | 1 (14.3) | 0 (0.0) | 6 (85.7) | 5 (71.4) | 2 (28.6) | 0 (0.0) |
| Radiation Therapist | **7** | 6 (85.7) | 1 (14.3) | 6 (85.7) | 1 (14.3) | 0 (0.0) | | 3 (42.9) | 3 (42.9) | 1 (14.3) | 4 (57.1) | 0 (0.0) | 0 (0.0) | 3 (42.9) | 0 (0.0) | 0 (0.0) | 7 (100.0) | 6 (85.7) | 0 (0.0) | 1 (14.3) |
| Mammographer | **6** | 6 (100.0) | 0 (0.0) | 0 (0.0) | 5 (83.3) | 1 (16.7) | | 0 (0.0) | 1 (16.7) | 5 (83.3) | 5 (83.3) | 0 (0.0) | 1 (16.7) | 0 (0.0) | 3 (50.0) | 0 (0.0) | 3 (50.0) | 5 (83.3) | 0 (0.0) | 1 (16.7) |
| Scientific/Research Officer | **6** | 6 (100.0) | 0 (0.0) | 4 (66.7) | 2 (33.3) | 0 (0.0) | | 3 (50.0) | 0 (0.0) | 3 (50.0) | 4 (66.7) | 1 (16.7) | 0 (0.0) | 1 (16.7) | 2 (33.3) | 1 (16.7) | 3 (50.0) | 6 (100.0) | 0 (0.0) | 0 (0.0) |
| Health/Medical Physicist | **5** | 5 (100.0) | 0 (0.0) | 2 (40.0) | 1 (20.0) | 2 (40.0) | | 3 (60.0) | 0 (0.0) | 2 (40.0) | 3 (60.0) | 0 (0.0) | 0 (0.0) | 2 (40.0) | 3 (60.0) | 0 (0.0) | 2 (40.0) | 5 (100.0) | 0 (0.0) | 0 (0.0) |
| Cardiology Health Professional | **4** | 4 (100.0) | 0 (0.0) | 2 (50.0) | 2 (50.0) | 0 (0.0) | | 2 (50.0) | 0 (0.0) | 2 (50.0) | 2 (50.0) | 2 (50.0) | 0 (0.0) | 0 (0.0) | 1 (25.0) | 1 (25.0) | 2 (50.0) | 4 (100.0) | 0 (0.0) | 0 (0.0) |
| Complementary Health Therapist | **4** | 4 (100.0) | 0 (0.0) | 0 (0.0) | 4 (100.0) | 0 (0.0) | | 0 (0.0) | 0 (0.0) | 4 (100.0) | 2 (50.0) | 0 (0.0) | 2 (50.0) | 0 (0.0) | 1 (25.0) | 0 (0.0) | 3 (75.0) | 4 (100.0) | 0 (0.0) | 0 (0.0) |
| Dental Prosthetist | **4** | 3 (75.0) | 1 (25.0) | 4 (100.0) | 0 (0.0) | 0 (0.0) | | 2 (50.0) | 2 (50.0) | 0 (0.0) | 1 (25.0) | 2 (50.0) | 0 (0.0) | 1 (25.0) | 0 (0.0) | 0 (0.0) | 4 (100.0) | 3 (75.0) | 0 (0.0) | 1 (25.0) |
| Orthotist/Prosthetist | **4** | 4 (100.0) | 0 (0.0) | 4 (100.0) | 0 (0.0) | 0 (0.0) | | 4 (100.0) | 0 (0.0) | 0 (0.0) | 4 (100.0) | 0 (0.0) | 0 (0.0) | 0 (0.0) | 0 (0.0) | 1 (25.0) | 3 (75.0) | 4 (100.0) | 0 (0.0) | 0 (0.0) |
| Dental Therapist | **3** | 2 (66.7) | 1 (33.3) | 1 (33.3) | 2 (66.7) | 0 (0.0) | | 0 (0.0) | 0 (0.0) | 3 (100.0) | 0 (0.0) | 1 (33.3) | 0 (0.0) | 2 66.7) | 1 (33.3) | 0 (0.0) | 2 (66.7) | 1 (33.3) | 0 (0.0) | 2 (66.7) |
| Respiratory Scientist | **3** | 3 (100.0) | 0 (0.0) | 2 (66.7) | 1 (33.3) | 0 (0.0) | | 2 (66.7) | 0 (0.0) | 1 (33.3) | 1 (33.3) | 2 (66.7) | 0 (0.0) | 0 (0.0) | 0 (0.0) | 3 (100.0) | 0 (0.0) | 2 (66.7) | 1 (33.3) | 0 (0.0) |
| Microbiologist | **2** | 2 (100.0) | 0 (0.0) | 2 (100.0) | 0 (0.0) | 0 (0.0) | | 1 (50.0) | 1 (50.0) | 0 (0.0) | 2 (100.0) | 0 (0.0) | 0 (0.0) | 0 (0.0) | 0 (0.0) | 2 (100.0) | 0 (0.0) | 2 (100.0) | 0 (0.0) | 0 (0.0) |
| Perfusionist | **2** | 2 (100.0) | 0 (0.0) | 2 (100.0) | 0 (0.0) | 0 (0.0) | | 1 (50.0) | 1 (50.0) | 0 (0.0) | 2 (100.0) | 0 (0.0) | 0 (0.0) | 0 (0.0) | 0 (0.0) | 0 (0.0) | 2 (100.0) | 2 (100.0) | 0 (0.0) | 0 (0.0) |
| Epidemiologist | **1** | 1 (100.0) | 0 (0.0) | 0 (0.0) | 1 (100.0) | 0 (0.0) | | 0 (0.0) | 0 (0.0) | 1 (100.0) | 1 (100.0) | 0 (0.0) | 0 (0.0) | 0 (0.0) | 0 (0.0) | 0 (0.0) | 1 (100.0) | 1 (100.0) | 0 (0.0) | 0 (0.0) |
| Nuclear Medicine Health Professional | **1** | 1 (100.0) | 0 (0.0) | 1 (100.0) | 0 (0.0) | 0 (0.0) | | 1 (100.0) | 0 (0.0) | 0 (0.0) | 1 (100.0) | 0 (0.0) | 0 (0.0) | 0 (0.0) | 0 (0.0) | 0 (0.0) | 1 (100.0) | 1 (100.0) | 0 (0.0) | 0 (0.0) |
| Total | **3967** | **3233 (81.5)** | **734 (18.5)** | **1325 (33.4)** | **2321 (58.5)** | **321 (8.1)** | | **1186 (29.9)** | **982 (24.8)** | **1799 (45.3)** | **1514 (38.2)** | **954 (24.0)** | **496 (12.5)** | **1003 (25.3)** | **1455 (36.7)** | **457 (11.5)** | **2050 (51.7)** | **2845 (71.7)** | **750 (18.9)** | **372 (9.4)** |

*AHPRA regulated profession
