# Supplementary material for: Are recent health, welfare and care graduates part of a rural and remote workforce solution? Evidence from Tasmania, Australia
Source: BMC Health Serv Res. 2024 May 21;24:652. doi: 10.1186/s12913-024-11087-9 (PMC11110370; doi:10.1186/s12913-024-11087-9)
Supplement: Supplementary file 3 — Supplementary Material 3. Additional File 3. Word document. Proportion of graduate suitable job advertisements by advertisement characteristic (n = 184). Detailed analysis of graduate suitable job advertisements by characteristics. [file 12913_2024_11087_MOESM3_ESM.docx]

Additional File 3. Proportion of graduate suitable job advertisements by advertisement characteristic (n=184)

| Profession | Adverts | Advertisement Characteristic | | | | | | | | | | | | | | | | | |
| --- | --- | --- | --- | --- | --- | --- | --- | --- | --- | --- | --- | --- | --- | --- | --- | --- | --- | --- | --- |
|  |  | **Positions** | | **Employer** | | | **Tenure** | | | **Hours** | | | | **Experience** | | | **Location** | | |
|  |  | Single | Multiple | Govt | Non-Govt | Not Specified | Permanent | Temporary/ Fixed Term | Mixed/Not Specified | Full-Time | Part- Time | Casual/  Locum | Mixed/Not Specified | Essential | Desirable | Not specified | MM2 | MM3-7 | Mixed/ Not Specified |
|  | **n** | **n (%)** | **n (%)** | **n (%)** | **n (%)** | **n (%)** | **n (%)** | **n (%)** | **n (%)** | **n (%)** | **n (%)** | **n (%)** | **n (%)** | **n (%)** | **n (%)** | **n (%)** | **n (%)** | **n (%)** | **n (%)** |
| Physiotherapist* | **49** | 42 (85.8) | 7 (14.3) | 4 (8.2) | 32 (65.3) | 13 (26.5) | 11 (22.4) | 6 (12.2) | 32 (65.3) | 19 (38.8) | 3 (6.1) | 4 (8.2) | 23 (46.9) | 2 (4.1) | 6 (12.3) | 41 (83.7) | 40 (81.6) | 5 (10.2) | 4 (6.1) |
| Multiple Professions Specified | **33** | 16 (48.5) | 17 (51.5) | 1 (3.0) | 26 (78.8) | 6 (18.2) | 0 (0.0) | 8 (24.2) | 25 (75.8) | 15 (45.5) | 2 (6.1) | 6 (18.2) | 10 (30.3) | 3 (9.1) | 10 (30.3) | 20 (60.6) | 24 (72.7) | 3 (9.1) | 6 (18.2) |
| Occupational Therapist* | **21** | 20 (95.2) | 1 (4.8) | 1 (4.8) | 14 (66.7) | 6 (28.6) | 1 (4.8) | 7 (33.3) | 13 (61.9) | 8 (38.1) | 1 (4.8) | 6 (28.6) | 6 (28.6) | 0 (0.0) | 4 (19.1) | 17 (81.0) | 18 (85.7) | 0 (0.0) | 3 (14.3) |
| Registered Nurse* | **15** | 10 (66.7) | 5 (33.3) | 1 (6.7) | 9 (60.0) | 5 (33.3) | 3 (20.0) | 5 (33.3) | 7 (46.7) | 5 (33.3) | 6 (40.0) | 1 (6.7) | 3 (20.0) | 2 (13.3) | 5 (33.3) | 8 (53.3) | 9 (60.0) | 2 (13.3) | 4 (26.7) |
| Podiatrist* | **12** | 10 (83.3) | 2 (16.7) | 1 (8.3) | 10 (83.3) | 1 (8.3) | 1 (8.3) | 2 (16.7) | 9 (75.0) | 8 (66.7) | 0 (0.0) | 0 (0.0) | 4 (33.3) | 1 (8.3) | 1 (8.3) | 10 (83.3) | 6 (50.0) | 3 (25.0) | 3 (25.0) |
| Exercise Physiologist | **7** | 7 (100.0) | 0 (0.0) | 0 (0.0) | 7 (100.0) | 0 (0.0) | 1 (14.3) | 0 (0.0) | 6 (85.7) | 4 (57.1) | 0 (0.0) | 0 (0.0) | 3 (42.9) | 0 (0.0) | 0 (0.0) | 7 (100.0) | 5 (71.4) | 2 (28.6) | 0 (0.0) |
| Sonographer/ Ultrasonographer | **6** | 5 (83.3) | 1 (16.7) | 0 (0.0) | 6 (100.0) | 0 (0.0) | 0 (0.0) | 1 (16.7) | 5 (83.3) | 1 (16.7) | 0 (0.0) | 0 (0.0) | 5 (83.3) | 0 (0.0) | 0 (0.0) | 6 (100.0) | 4 (66.7) | 2 (33.3) | 0 (0.0) |
| Pharmacist* | **5** | 4 (80.0) | 1 (20.0) | 0 (0.0) | 4 (80.0) | 1 (20.0) | 2 (40.0) | 1 (20.0) | 2 (40.0) | 3 (60.0) | 2 (40.0) | 0 (0.0) | 0 (0.0) | 2 (40.0) | 0 (0.0) | 3 (60.0) | 4 (80.0) | 1 (20.0) | 0 (0.0) |
| Midwife* | **5** | 1 (20.0) | 4 (80.0) | 1 (20.0) | 4 (80.0) | 0 (0.0) | 4 (80.0) | 1 (20.0) | 0 (0.0) | 0 (0.0) | 0 (0.0) | 0 (0.0) | 5 (100.0) | 0 (0.0) | 0 (0.0) | 5 (100.0) | 4 (80.0) | 0 (0.0) | 1 (20.0) |
| Welfare Worker | **4** | 3 (75.0) | 1 (25.0) | 0 (0.0) | 3 (75.0) | 1 (25.0) | 1 (25.0) | 2 (50.0) | 1 (25.0) | 1 (25.0) | 1 (25.0) | 0 (0.0) | 2 (50.0) | 0 (0.0) | 2 (50.0) | 2 (50.0) | 3 (75.0) | 1 (25.0) | 0 (0.0) |
| Enrolled Nurse* | **4** | 2 (50.0) | 2 (50.0) | 3 (75.0) | 1 (25.0) | 0 (0.0) | 1 (25.0) | 2 (50.0) | 1 (25.0) | 0 (0.0) | 3 (75.0) | 0 (0.0) | 1 (25.0) | 0 (0.0) | 0 (0.0) | 4 (100.0) | 3 (75.0) | 1 (25.0) | 0 (0.0) |
| Optometrist* | **4** | 3 (75.0) | 1 (25.0) | 0 (0.0) | 4 (100.0) | 0 (0.0) | 0 (0.0) | 0 (0.0) | 4 (100.0) | 3 (75.0) | 0 (0.0) | 0 (0.0) | 1 (25.0) | 0 (0.0) | 1 (25.0) | 3 (75.0) | 2 (50.0) | 0 (0.0) | 2 (50.0) |
| Speech Pathologist | **3** | 3 (100.0) | 0 (0.0) | 0 (0.0) | 2 (66.7) | 1 (33.3) | 0 (0.0) | 0 (0.0) | 3 (100.0) | 1 (33.3) | 0 (0.0) | 0 (0.0) | 2 (66.7) | 1 (33.3) | 0 (0.0) | 2 (66.7) | 2 (66.7) | 0 (0.0) | 1 (33.3) |
| Radiographer* | **3** | 3 (100.0) | 0 (0.0) | 0 (0.0) | 2 (66.7) | 1 (33.3) | 0 (0.0) | 0 (0.0) | 3 (100.0) | 3 (100.0) | 0 (0.0) | 0 (0.0) | (0.0) | 0 (0.0) | 0 (0.0) | 3 (100.0) | 3 (100.0) | 0 (0.0) | 0 (0.0) |
| Paramedic* | **3** | 0 (0.0) | 3 (100.0) | 3 (100.0) | 0 (0.0) | 0 (0.0) | 0 (0.0) | 2 (66.7) | 1 (33.3) | 2 (66.7) | 0 (0.0) | 0 (0.0) | 1 (33.3) | 0 (0.0) | 0 (0.0) | 3 (100.0) | 0 (0.0) | 0 (0.0) | 3 (100.0) |
| Psychologist* | **2** | 1 (50.0) | 1 (50.0) | 0 (0.0) | 2 (100.0) | 0 (0.0) | 0 (0.0) | 0 (0.0) | 2 (100.0) | 0 (0.0) | 1 (50.0) | 0 (0.0) | 1 (50.0) | 0 (0.0) | 1 (50.0) | 1 (50.0) | 2 (100.0) | 0 (0.0) | 0 (0.0) |
| Nursing Support Worker | **2** | 2 (100.0) | 0 (0.0) | 2 (100.0) | 0 (0.0) | 0 (0.0) | 0 (0.0) | 2 (100.0) | 0 (0.0) | 0 (0.0) | 0 (0.0) | 2 (100.0) | (0.0) | 1 (50.0) | 0 (0.0) | 1 (50.0) | 1 (50.0) | 1 (50.0) | 0 (0.0) |
| Child and Family/Youth Justice Health Professional | **1** | 1 (100.0) | 0 (0.0) | 0 (0.0) | 1 (100.0) | 0 (0.0) | 1 (100.0) | 0 (0.0) | 0 (0.0) | 0 (0.0) | 1 (100.0) | 0 (0.0) | (0.0) | 0 (0.0) | 0 (0.0) | 1 (100.0) | 1 (100.0) | 0 (0.0) | 0 (0.0) |
| Dietitian | **1** | 1 (100.0) | 0 (0.0) | 0 (0.0) | 1 (100.0) | 0 (0.0) | 0 (0.0) | 0 (0.0) | 1 (100.0) | 1 (100.0) | 0 (0.0) | 0 (0.0) | (0.0) | 0 (0.0) | 0 (0.0) | 1 (100.0) | 1 (100.0) | 0 (0.0) | 0 (0.0) |
| Environmental/ Public Health Officer | **1** | 1 (100.0) | 0 (0.0) | 1 (100.0) | 0 (0.0) | 0 (0.0) | 0 (0.0) | 0 (0.0) | 1 (100.0) | 1 (100.0) | 0 (0.0) | 0 (0.0) | (0.0) | 0 (0.0) | 0 (0.0) | 1 (100.0) | 0 (0.0) | 1 (100.0) | 0 (0.0) |
| Hospital/Medical Scientist | **1** | 1 (100.0) | 0 (0.0) | 0 (0.0) | 0 (0.0) | 1 (100.0) | 0 (0.0) | 0 (0.0) | 1 (100.0) | 0 (0.0) | 1 (100.0) | 0 (0.0) | (0.0) | 0 (0.0) | 0 (0.0) | 1 (100.0) | 1 (100.0) | 0 (0.0) | 0 (0.0) |
| Health/Medical Physicist | **1** | 1 (100.0) | 0 (0.0) | 0 (0.0) | 0 (0.0) | 1 (100.0) | 1 (100.0) | 0 (0.0) | 0 (0.0) | 1 (100.0) | 0 (0.0) | 0 (0.0) | (0.0) | 1 (100.0) | 0 (0.0) | 0 (0.0) | 1 (100.0) | 0 (0.0) | 0 (0.0) |
| Dental Therapist | **1** | 1 (100.0) | 0 (0.0) | 0 (0.0) | 1 (100.0) | 0 (0.0) | 0 (0.0) | 0 (0.0) | 1 (100.0) | 0 (0.0) | 1 (100.0) | 0 (0.0) | 0 (0.0) | 0 (0.0) | 0 (0.0) | 1 (100.0) | 1 (100.0) | 0 (0.0) | 0 (0.0) |
| Total | **184** | **138 (75.0)** | **46 (25.0)** | **18 (9.8)** | **129 (70.1)** | **37 (20.1)** | **27 (14.7)** | **38 (20.7)** | **115 (62.5)** | **76 (41.3)** | **22 (12.0)** | **19 (10.3)** | **67 (36.4)** | **13 (7.1)** | **30 (16.3)** | **141 (76.6)** | **135 (73.4)** | **22 (12.0)** | **19 (10.3)** |

*AHPRA regulated profession
